# Transition of Carrier Transport Behaviors with Temperature in Phosphorus-Doped Si Nanocrystals/SiO_2_ Multilayers

**DOI:** 10.1186/s11671-016-1561-z

**Published:** 2016-07-26

**Authors:** Mingqing Qian, Dan Shan, Yang Ji, Dongke Li, Jun Xu, Wei Li, Kunji Chen

**Affiliations:** National Laboratory of Solid State Microstructures, School of Electronic Science and Engineering and Collaborative Innovation Center of Advanced Microstructures, Nanjing University, Hankou Road 22, Nanjing City, Jiangsu Province 210093 China

**Keywords:** Phosphorus-doped, Si nanocrystals/SiO_2_ multilayers, Carrier transport

## Abstract

High-conductive phosphorus-doped Si nanocrystals/SiO_2_(nc-Si/SiO_2_) multilayers are obtained, and the formation of Si nanocrystals with the average crystal size of 6 nm is confirmed by high-resolution transmission electron microscopy and Raman spectra. The temperature-dependent carrier transport behaviors of the nc-Si/SiO_2_ films are systematically studied by which we find the shift of Fermi level on account of the changing P doping concentration. By controlling the P doping concentration in the films, the room temperature conductivity can be enhanced by seven orders of magnitude than the un-doped sample, reaching values up to 110 S/cm for heavily doped sample. The changes from Mott variable-range hopping process to thermally activation conduction process with the temperature are identified and discussed.

## Background

Nanocrystalline Si (nc-Si) materials have been extensively studied because of their novel properties and their potential applications in future electronic and optoelectronic devices [1, 2]. For example, the SiO_2_/nc-Si/SiO_2_ sandwiched structures can be used as a floating gate to develop the non-volatile memories [3]. It has also been reported that nc-Si-based materials can be potentially used in light-emitting devices as well as next generation solar cells [4–6]. However, most of the published work focused on the un-doped nc-Si materials. In order to further improve the devices performance, it is necessary to study the doping effect in nc-Si materials, since it can effectively control the electronic structures and properties of semiconductors [7, 8]. It was reported that the doping of P and B in nc-Si is difficult due to Self-Purification effect, which is quite different from their bulk counterpart [8–10]. The further work pointed out that P atoms tend to stay in the core, and B atoms prefer to locate near the surface for the H-passivated Si nanocrystals [11]. While Pi et al. studied the doping of P and B in H-passivated free-standing Si nanocrystals and they found that P atoms resides at or near the surface of the Si nanocrystals while B is in the core region, which seems contrary to the theoretical results [12]. The optical properties and electronic structures and conductivity of P-doped nc-Si films have also been studied. Fujii et al. found that photoluminescence in nc-Si can be enhanced by P doping [13]. Stegner et al. found that the conductivity of free-standing Si nanocrystals with the average size of 30 nm increased with the doping concentrations and exhibited a decrease of temperature dependence in the temperature range of 100–300 K [14]. However, the experimental study on temperature-dependent conduction behaviors in a wide temperature range is still lack for doped nc-Si films though it is important to help one understand the carrier transport process in nanocrystalline Si films.

In our previous work, we fabricated the P-doped nc-Si/SiO_2_ multilayers by thermally annealing P-doped amorphous Si/SiO_2_ stacked structures. It was found that part of the P impurities can be located at the inner sites of nc-Si substitutionally besides the passivation of the Si dangling bonds at the interface sites of the nc-Si dots [15]. P doping also affected the electrical structures of nc-Si and induced the subband light emitting in the infrared light region [15, 16]. In the present work, temperature-dependent carrier transport processes in P-doped nc-Si/SiO_2_ multilayers are systematically studied in a temperature range of 40–660 K. It is found that the room temperature conductivity of P-doped nc-Si/SiO_2_ multilayers is significantly enhanced due to the doping effect. By controlling the doping concentrations, the carrier concentrations can be manipulated effectively. Based on the temperature-dependent conductivity measurements, the carrier transport mechanisms are discussed, which exhibit different behaviors with the temperature as well as the doping concentrations.

## Methods

The un-doped and P-doped hydrogenated amorphous Si/SiO_2_ stacked structures were fabricated on p-type Si substrates (1–3 Ω/cm) and quartz substrates in a conventional plasma-enhanced chemical vapor deposition (PECVD) system. SiH_4_ with a flow rate of 5 sccm was used to deposit the a-Si (amorphous) layer while the hydrogen diluted phosphine (PH_3_, 1 % in H_2_) with a various flow rate from 0 sccm to 3 sccm was simultaneously introduced to get different doping concentrations. Here, we use the nominal gas doping ratio of [PH_3_]/[SiH_4_] to denote the samples. The in situ plasma oxidation was subsequently performed using O_2_ with a flow rate of 20 sccm. Both the a-Si:H deposition time and the plasma oxidation time was kept at 90 s. The deposition and oxidation process was alternatively repeated for 15 times to get the multilayered structures. During the deposition process, the RF power and substrate temperature was 50 W and 250 °C, respectively. The as-deposited samples were then dehydrogenated at 450 °C followed by high temperature annealing at 1000 °C in N_2_ ambient for 1 h to nanocrystallize the a-Si:H layers.

Raman scattering spectra were performed by a Jobin Yvon Horiba HR800 spectrometer with an Ar^+^ laser with the wavelength of 514 nm as excitation light source. The high-resolution transmission electron microscopy (TEM) images were observed by a TECNAI G2F20 FEI high-resolution transmission electron microscopy. The X-ray photoelectron spectroscopy (XPS) measurements were performed with a PHI 5000 Versa Probe system, and the composition signals of Si and P in the nc-Si layer were detected after Ar^+^ etching for 100 s with the etching depth of 10 nm. The C 1 s line at 285 eV has been used as a reference to rectify the charge shift of the binding energies. The conductivity and Hall mobility were obtained by temperature-dependent Hall measurements using van der Pauw (VDP) geometry with the LakeShore 8400 Hall effect measurement system. The temperature of the Hall measurement ranges from 40 to 660 K. The samples were prepared with coplanar Al electrodes on the four corners of the film by vacuum thermal evaporation followed by a 400 °C alloying treatment of 30 mins to achieve the ohmic contacts which is confirmed by the linear current–voltage relationship.

## Results and Discussions

### Structural Properties

**Fig. 1:**
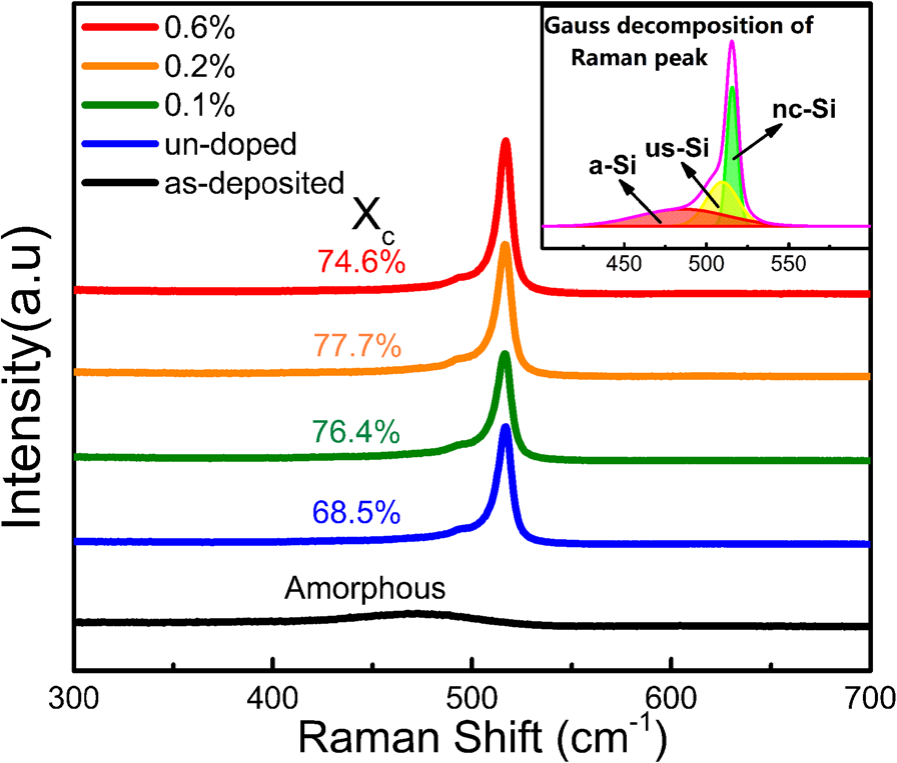
Raman spectra of samples with various P doping concentrations at the annealing temperature of 1000 °C. The *inset* is the Gauss decomposition of Raman peak

Raman spectroscopy was used to identify the formation of Si nanocrystals. Figure 1 shows the Raman spectra of the P-doped Si/SiO_2_ multilayers after 1000 °C annealing at various nominal doping concentrations. For the as-deposited sample, only a weak and broad Raman band centered at 480 cm^−1^ can be detected, which is attributed to the transverse-optical (TO) vibration mode of amorphous Si–Si bands. Meanwhile in the samples that undergo annealing procedure, a sharp peak around 517 cm^−1^ emerges which indicates the formation of nanocrystalline Si phases. The crystalline volume fractions (*X*_*c*_) of the samples after annealing have been estimated with the formula: ${X}_c=\frac{\mathrm{Ic}}{\mathrm{Ic}+0.88\mathrm{I}\mathrm{a}}$ [17] by the deconvolution of each Raman spectrum into three Gaussian components corresponding to nanocrystalline (nc-Si), amorphous (a-Si), and the intermediate ultra-small nanocrystalline (us-Si) component, as shown in the inset of Fig. 1, where *Ic* is the crystalline part of the integrated Raman scattering intensity and *Ia* is the amorphous part of the integrated Raman scattering intensity. The average size of nc-Si is about 5.8 nm calculated by the phonon confinement model [18]. It is found that *X*_*c*_ increases with the doping concentration when the doping level is low, which indicates that P dopants can promote the crystallization process of Si [19]. This is because P dopants tend to first passivate the Si dangling bonds which helps increase the order of the film and then locate at the substitutional sites inside the Si nanocrystals as P_4_^0^ which can lower the formation energy of Si nanocrystals [15, 20]. However, when the P concentration is further increased, the crystalline fraction *X*_*c*_ drops a little bit. This may because more P dopants incorporated into the films and cause damage to the crystal lattice. And it forms the defect states which may result in the degradation of crystallinity [15].

**Fig. 2:**
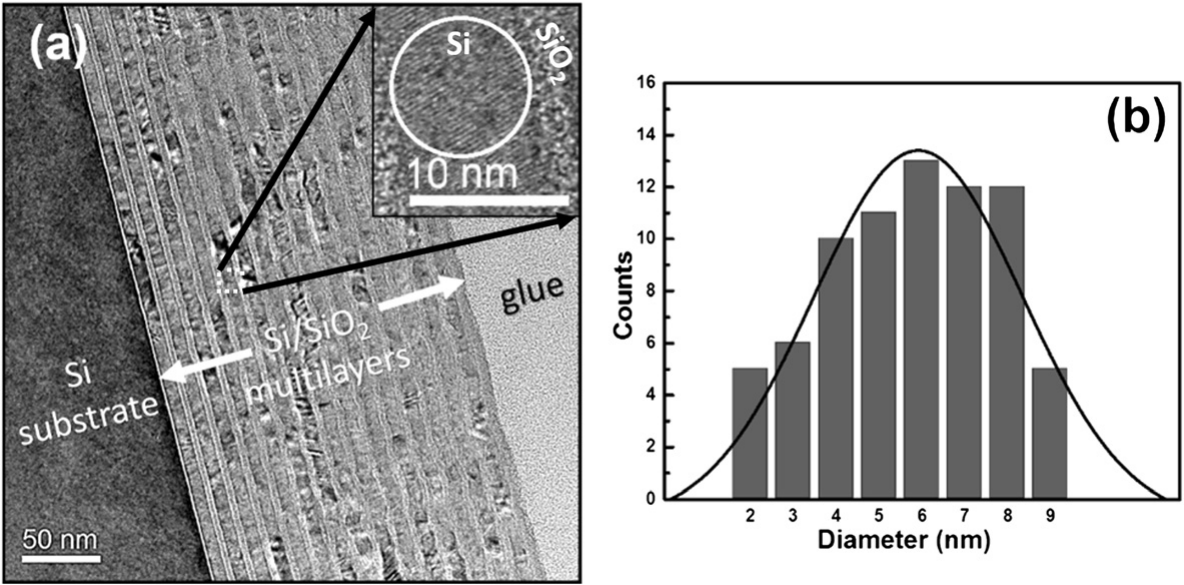
**a** Cross-section TEM micrographs with low and high magnifications for the nominal P concentration of 0.2 % nc-Si/SiO_2_ multilayers after 1000 °C annealing. **b** Size distribution of the nc-Si dots in the doped nc-Si/SiO_2_ film

The multilayered structures after 1000 °C annealing were investigated using the cross-sectional TEM observations. Figure 2a shows the TEM image of nc-Si/SiO_2_ multilayers with the nominal P doping ratio of 0.2 % after annealing. The periodically layered structures are well kept, and the interfaces are still smooth after high temperature annealing. The thickness of the nc-Si layer is about 8.5 nm, and the thickness of SiO_2_ layer is about 2–3 nm. The formation of Si nanocrystals can be further identified in the high-resolution TEM image as given in the inset of Fig. 2a, which exhibits the crystallized Si with the size about 8–10 nm. The crystal lattice can be clearly identified and the crystalline inter-planar spacing of formed Si nanocrystals is about 0.31 nm, which corresponds to the Si (111) crystalline orientation [5]. The size distribution of nc-Si dots is evaluated by a statistic on 74 nc-Si dots, as shown in Fig. 2b. The average diameter of nc-Si dots is about 6 nm, and the size deviation is about 2.2 nm which is consistent with the Raman result.

**Fig. 3:**
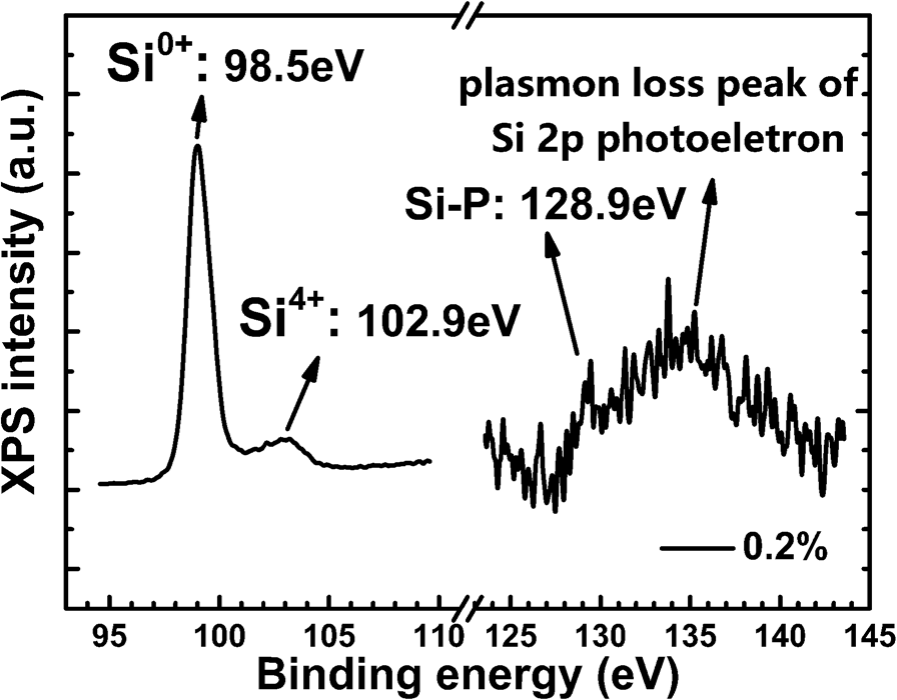
X-ray photoelectron spectroscopy (XPS) of the 1000 °C annealed samples with the nominal doping concentration of 0.2 %

In order to testify the existence of P in the nc-Si layer, we performed the X-ray photoelectron spectroscopy (XPS) measurements on the 0.2 % P-doped samples after etching 100 s. As shown in Fig. 3, a sharp peak at 98.5 eV and a small peak at 102.9 eV appear, which correspond to the Si–Si (Si^0+^) bond and the Si–O (Si^4+^) bond, respectively [21]. In our previous work, we detected the depth-dependent XPS spectra and found out the periodically changes of the signals from Si–Si (Si^0+^) bond and Si–O (Si^4+^) bond [15]. The ratio of the two signals suggests that the detected position is in the nc-Si layer. Meanwhile in the P 2p spectrum, a signal at 128.9 eV corresponding to the P–Si bond can be identified, which implies that the P atoms exist in the nc-Si layer in the form of P–Si bond after high temperature annealing, while the broad peak centered at 133 eV is the plasmon loss peak of Si 2p photoelectron [21]. We have also used the low-temperature electron spin resonance (ESR) measurements to study the P-doped nc-Si/SiO_2_ multilayers in our previous work, and it was found that a clear ESR signal of the conduction electrons can be observed in the samples which demonstrated that part of the P impurities indeed occupies the inner sites of nc-Si substitutionally and provide the conduction electrons after ionization [15].

### Electrical Properties

**Fig. 4:**
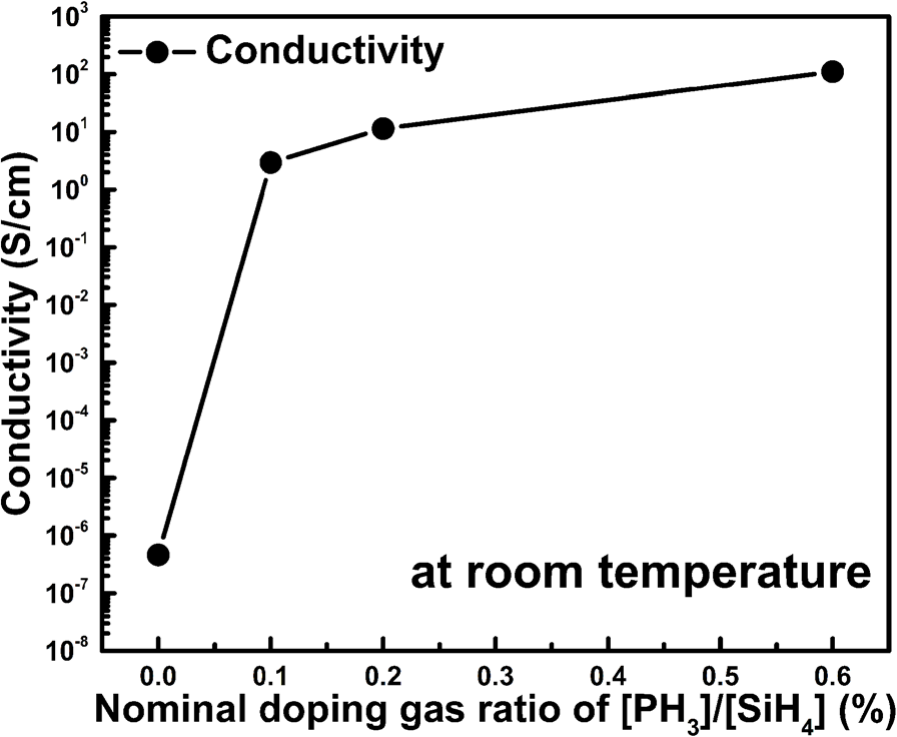
The conductivity tendency of the various P-doped nc- Si/SiO_2_ multilayers at room temperature

**Fig. 5:**
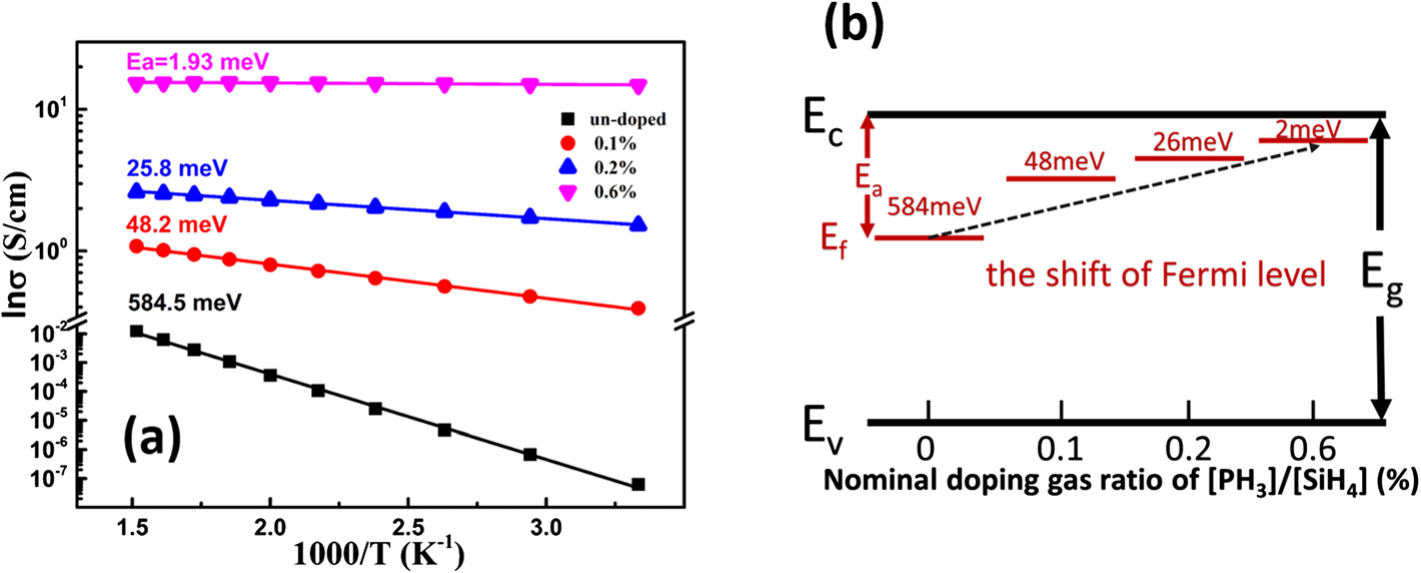
**a** The lnσ-1/T plot with different P doping ratios. The value listed on each line is the corresponding conductivity activation energy *E*_*a*_. **b** The schematic drawing of the shift of Fermi level

In order to understand the doping effect on the electrical properties of P-doped nc-Si/SiO_2_ multilayers, temperature-dependent conductivities were measured first in a temperature ranged from 300 to 660 K. As shown in Fig. 4, it is found that the conductivity increases monotonically with the P doping concentration and the conductivity of doped samples can be as high as 110 S/cm at room temperature which is enhanced by seven orders of magnitude than that of the un-doped one. Furthermore, it is found that both the doped and the un-doped samples annealed at 1000 °C exhibit linear relationship in the lnσ-1/T plot in the temperature region 300–660 K given in Fig. 5a, which matches the Arrhenius relationship between dark conductivity *σ* and the temperature *T*, described as the formula: *σ* = *σ*_0_exp(−*E*_*a*_/*k*_*B*_*T*), where, *σ*_0_ is the pre-exponential factor of conductivity, *E*_*a*_ is the conductivity activation energy, and *k*_*B*_ is the Boltzmann’s constant [22]. It is indicated that the transport behavior of doped nc-Si/SiO_2_ above room temperature is thermal activation conduction mechanism. The activation energy *E*_*a*_ can be deduced through Arrhenius plots by the slope of the linear fit, which is related to the energy difference between the Fermi level and the bottom of conduction band in *n*-type semiconductor. The deduced conductivity activation energy *E*_*a*_ is given in Fig. 5a, b. For un-doped samples, *E*_*a*_ is about 0.58 eV which implies the Fermi level locates at the mid gap of the nc-Si. With increasing the P doping ratio, the *E*_*a*_ decreased rapidly, which indicates that the Fermi level shifts to the conduction band due to the doping effect as shown in the Fig. 5b. It is interesting to find that when the nominal P doping ratio is high enough (~0.6 %), the activation energy *E*_*a*_ is very small, which means that the Fermi level should be closed to the bottom of the conduction band. As a consequence, the conductivity is almost unchanged with the temperature.

The significantly increased room temperature conductivity by seven orders of magnitude after doping can be attributed to the increased carrier concentrations after P doping. According to the formula: ${n}_0={N}_c \exp \left(\frac{E_f-{E}_c}{k_oT}\right)$ for non-degenerate samples and ${n}_0={N}_c\frac{2}{\sqrt{\uppi}}{\mathrm{F}}_{1/2}\left(\frac{E_f-{E}_c}{k_oT}\right)$ for degenerate samples (doping ratio ~0.6 %), where *n*_0_ is the carrier concentration, *N*_*c*_ is the effective density of conduction band, *E*_*f*_ is the Fermi level, *E*_*c*_ is the conduction band, and F_1/2_ refers to the Fermi integral function, we can roughly estimate that the carrier concentration of the doped samples is seven orders of magnitude higher than the un-doped one if we assume that the Fermi level shifts from 0.58 eV to the bottom of conduction band *E*_*c*_ after P doping as shown in Fig. 5b. Since we measured the Hall mobility and room conductivity of samples with and without doping, one can also estimate the carrier concentrations again from the formula: *σ* = nμq. It is found that the mobility is about 1.2 cm^2^/V s for the un-doped sample, and it is increased to 9.8 cm^2^/V s for sample with doping gas ratio of 0.6 %. The calculated carrier concentration is 2.4 × 10^12^ and 7.1 × 10^19^ cm^−3^ for the un-doped sample and the sample with the doping gas ratio of 0.6 %, which is well consistent with the result estimated from the shift of *E*_*a*_. It is clearly demonstrated that P dopants can be incorporated into the nc-Si dots to provide conduction electrons to control the conductivity.

We also measured the temperature-dependent conductivities in the low temperature range (from 40 K to room temperature). For the un-doped samples, the resistivity is quite high so that the conductivity in the low temperature range is difficult to be measured. According to Stegner et al., the increase of the Si nanocrystals’ doping concentration results in a decrease of the conductivity temperature dependence in the temperature range of 100–300 K [14]. In our case, we also found that the conductivity data of the doped nc-Si/SiO_2_ multilayers obtained below the room temperature (300 K) cannot be well described by the Arrhenius relationship which suggests different mechanisms dominating the carrier transport processes.

**Fig. 6:**
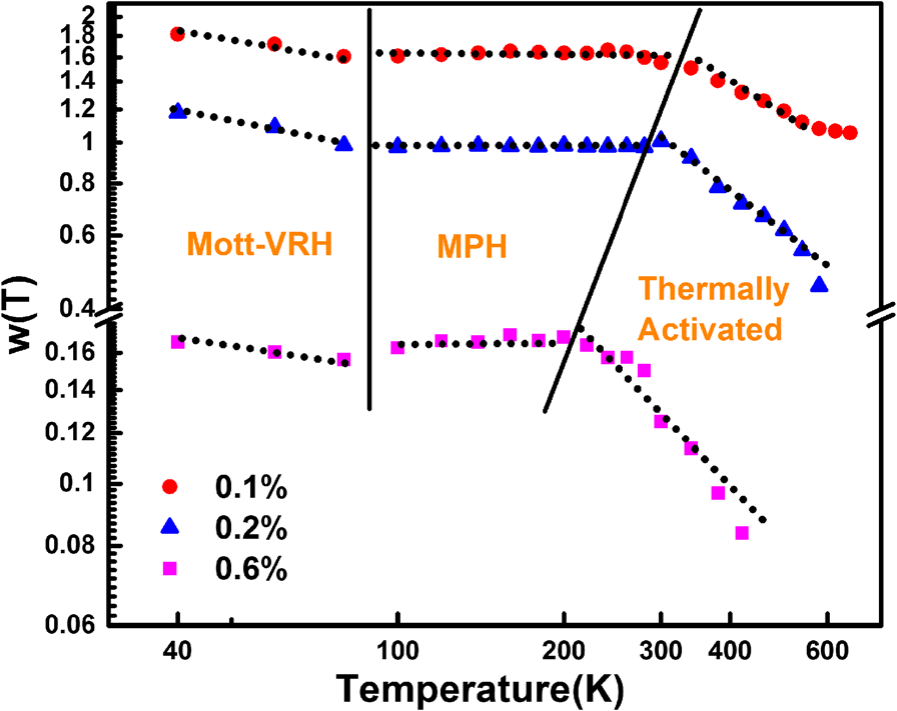
The reduced activation energy *w*(*T*) plotted against *T* on a log–log scale for samples with the nominal P concentration of 0.1, 0.2, and 0.6 %

In order to extract information about the transport behavior of the doped nc-Si multilayers at low temperature, the reduced activation energy,$w(T)=\frac{d\left( \ln \sigma \right)}{d\left( \ln T\right)}$, has been introduced and plotted against *T* on a log–log scale over the entire temperature range (40–660 K) [22, 23]. It is found that three different temperature regimes can be identified as shown in Fig. 6, which is different from the result of intrinsic and Boron-doped nc-Si materials reported by Concari et al. that only one transport mechanism, Mott variable-range hopping (Mott-VRH) occurs at the temperature range of 270 to 450 K [24]. The reduced activation energy *w*(*T*), appears to be a straight line with a slope ≈ 1 at the high temperature region, which refers to the thermally activated transport mechanism as we discussed before. The span of zero slope from room temperature down to *T* = 80 K refers to a power–law temperature dependence of conductivity (*σ* = T^γ^), which indicates the multiple phonon hopping (MPH) conduction mechanisms due to weak carrier-phonon interactions. Within this temperature range, the electrons are weakly localized and preferentially coupled to the phonons whose wavelengths are close to its localization length [25]. Therefore, these localized electrons can transport between the deep localized states originated from the defect states or grain boundaries with the multiple phonon hopping process. It was suggested that the MPH process only works at the temperature larger than the phonon’s characteristic temperature (~100 K) [26]. The similar MPH conduction mechanism has also been reported in amorphous and nanocrystalline semiconductors [25–27].

However, when the temperature is low enough, the temperature-dependent behaviors of conductivity are changed again and no longer dominated by the MPH process. As shown in Fig. 6, the slope = 0.25 of the *w*(*T*) − *T* plots below 80 K is identified which indicates the Mott-VRH conduction mechanism following the formula: $\sigma ={\sigma}_0 \exp \left[-{\left(\frac{T_o}{T}\right)}^{1/4}\right]$ [28]. It is reasonable since the ability of electrons utilizing multiple phonons for hopping is limited due to the freeze out of acoustic phonons as the temperature goes down to 80 K. According to Mott’s theory, the low temperature transport is due to the tunneling of carriers from occupied to unoccupied localized states [28]. The Mott-VRH conduction can be observed in un-doped and lightly doped microcrystalline silicon (μc-Si) films at low temperature as well [29–31]. It is found that with increasing the P doping concentrations, the temperature at which the conduction mechanism changing from MPH to thermally activated conduction becomes lower as shown in Fig. 6, for example, it is about 300 K for sample with doping ratio of 0.1 % and it becomes 200 K for sample with doping ratio of 0.6 %. This can be explained as the Fermi level shifts to the conduction band, thus more electrons can easily occupy the sites in conduction band and the thermally activated conduction process plays an important role in carrier transport behaviors even at the low temperature.

V. Osinniy et al. studied the temperature-dependent charge-carrier transport in un-doped nc-Si/SiO_2_ multilayer structures by evaporating Au layer on top and back side of the samples. It was indicated that the carrier transport can be well described by a combination of phonon-assisted and direct tunneling mechanisms since they measured the vertical electrical transport process where the SiO_2_ barriers play the crucial role in the current–voltage relationship. They also found that the carrier transport is thermally activated, but the resistivity curve was characterized by two slopes which means the change of the carrier transport mechanism though they did not discuss it in detail [32]. In our case, we used the co-planar geometrics to study the temperature-dependent Hall effect in P-doped nc-Si/SiO_2_ multilayers deposited on quartz substrates. Therefore, the studied electrical transport process mainly occurred in the planar nc-Si layers, and dopants have strong influences on the conduction process instead of SiO_2_ layers. The temperature-dependent conductivity indicated that the different transport mechanism from Mott-VRH conduction to thermally activated transport mechanism dominated transport process in the various temperature ranges since P doping can provide the electrons and introduce more defect states in nc-Si [15], and they may play important roles in the transport process especially at the low temperature.

## Conclusions

In summary, un-doped and P-doped nc-Si/SiO_2_ multilayers are formed by 1000 °C thermal annealing the corresponding hydrogenated amorphous Si/SiO_2_ stacked structures. It is found that the crystallinity is increased for samples with high doping concentrations, and the average size of formed Si nanocrystals is about 6 nm. The temperature-dependent conductivity with the investigation temperature above 300 K indicates the thermal activation conduction behavior dominating the carrier transport process. The gradual decrease of conductivity activation energy with the doping concentrations suggests that the shift of the Fermi level to the bottom of the conduction band and the corresponding room temperature conductivity is as high as 110 S/cm which is enhanced by seven orders of magnitude compare with that of the un-doped one. Furthermore, the temperature-dependent conductivity measurements in the low temperature range suggest that the Mott-VRH conduction mechanism dominates the transport process in doped samples at very low temperature, and MPH conduction mechanism appears when the investigation temperature increases to about 80 K. Our results demonstrate that high conductive nc-Si can be obtained after P doping, and the carrier transport mechanism depends both on the temperature and the doping levels.

## Abbreviations

nc-Si/SiO_2_: Si nanocrystals/SiO_2_; a-Si: amorphous Si; PECVD: plasma enhanced chemical vapor deposition; TEM: transmission electron microscopy; XPS: X-ray photoelectron spectroscopy; Mott-VRH: Mott variable-range hopping; MPH: multiple phonon hopping; ESR: electron spin resonance; μc-Si: microcrystalline silicon

## References

[1] Ray SK, Maikap S, Banerjee W, Das S (2013). Nanocrystals for silicon-based ligh-emitting and memory devices. J Phys D Appl Phys.

[2] Kang ZH, Liu Y, Lee ST (2011). Small-sized silicon nanoparticles: new nanolights and nanocatalysts. Nanoscale.

[3] Lien YC, Shieh JM, Huang WH, Tu CH, Wang C, Shen CH, Dai BT, Pan CL, Hu CM, Yang FL (2012). Fast programming metal-gate Si quantum dot nonvolatile memory using green nanosecond laser spike annealing. Appl Phys Lett.

[4] Mu W, Zhang P, Xu J, Sun S, Xu J, Li W, Chen K (2014). Direct-current and alternating-current driving Si quantum dots-based light emitting device. IEEE J Sel Top Quant.

[5] Cao Y, Lu P, Zhang X, Xu J, Xu L, Chen K (2014). Enhanced photovoltaic property by forming pin structures containing Si quantum dots/SiC multilayers. Nanoscale Res Lett.

[6] Löper P, Stüwe D, Künle M, Bivour M, Reichel C, Neubauer R, Schnabel M, Hermle M, Eibl O, Janz S (2012). A membrane device for substrate-free photovoltaic characterization of quantum dot based p-i-n solar cells. Adv Mater.

[7] Chen XY, Shen WZ, He YL (2005). Enhancement of electron mobility in nanocrystalline silicon/crystalline silicon heterostructures. J Appl Phys.

[8] Norris DJ, Efros AL, Erwin SC (2008). Doped nanocrystals. Science.

[9] Dalpian GM, Chelikowsky JR (2006). Self-purification in semiconductor nanocrystals. Phys Rev Lett.

[10] Chan T-L, Kwak H, Eom J-H, Zhang SB, Chelikowsky JR (2010). Self-purification in Si nanocrystals: An energetics study. Phys Rev B.

[11] Xu Q, Luo JW, Li SS, Xia JB, Li JB, Wei SH (2007). Chemical trends of defect formation in Si quantum dots: the case of group-III and group-V dopants. Phys Rev B.

[12] Pi XD, Gresback R, Liptak RW, Campbell SA, Kortshagen U (2008). Doping efficiency, dopant location, and oxidation of Si nanocrystals. Appl Phys Lett.

[13] Fujii M, Mimura A, Hayashi S, Yamamoto K (1999). Photoluminescence from Si nanocrystals dispersed in phosphosilicate glass thin films: improvement of photoluminescence efficiency. Appl Phys Lett.

[14] Stegner AR, Pereira RN, Klein K, Lechner R, Dietmueller R, Brandt MS, Stutzmann M, Wiggers H (2008). Electronic transport in phosphorus-doped silicon nanocrystal networks. Phys Rev Lett.

[15] Lu P, Mu W, Xu J, Zhang X, Zhang W, Li W, Xu L, Chen K (2016). Phosphorus doping in Si nanocrystals/SiO2 msultilayers and light emission with wavelength compatible for optical telecommunication. Sci Rep.

[16] Shieh J-M, Yu W-C, Huang JY, Wang C-K, Dai B-T, Jhan H-Y, Hsu C-W, Kuo H-C, Yang F-L, Pan C-L (2009). Near-infrared silicon quantum dots metal-oxide-semiconductor field-effect transistor photodetector. Appl Phys Lett.

[17] Tsu R (1982). Critical volume fraction of crystallinity for conductivity percolation in phosphorus-doped Si:F:H alloys. Appl Phys Lett.

[18] Campbell I, Fauchet PM (1986). The effects of microcrystal size and shape on the one phonon Raman spectra of crystalline semiconductors. Solid State Commun.

[19] Gullanar MH, Zhang YH, Chen H, Wei WS, Xu GY, Wang TM, Cui RQ, Shen WZ (2003). Effect of phosphorus doping on the structural properties in nc-Si:H thin films. J Cryst Growth.

[20] Street RA (1982). Doping and the Fermi energy in amorphous silicon. Phys Rev Lett.

[21] Ying WB, Mizokawa Y, Kamiura Y, Kawamoto K, Yang WY (2001). The chemical composition changes of silicon and phosphorus in the process of native oxide formation of heavily phosphorus doped silicon. Appl Surf Sci.

[22] Das D, Sain B (2013). Electrical transport phenomena prevailing in undoped nc-Si/a-SiNx:H thin films prepared by inductively coupled plasma chemical vapor deposition. J Appl Phys.

[23] Zabrodskii AG (2001). The Coulomb gap: the view of an experimenter. Philos Mag.

[24] Concari SB, Buitrago RH (2004). Hopping mechanism of electric transport in intrinsic and p-doped nanocrystalline silicon thin films. J Non-Cryst Solids.

[25] Shimakawa K (1989). Multiphonon hopping of electrons on defect clusters in amorphous germanium. Phys Rev B.

[26] Wienkes LR, Blackwell C, Kakalios J (2012). Electronic transport in doped mixed-phase hydrogenated amorphous/nanocrystalline silicon thin films. Appl Phys Lett.

[27] Shimakawa K, Miyake K (1989). Hopping transport of localized π electrons in amorphous carbon films. Phys Rev B.

[28] Mott NF (1969). Philos Mag.

[29] Brenot R, Vanderhaghen R, Drévillon B, Cabarrocas PR i, Rogel R, Mohammed-Brahim T (2001). Transport mechanisms in hydrogenated microcrystalline silicon. Thin Solid Films.

[30] Liu F, Zhu M, Feng Y, Han Y, Liu J (2001). Electrical transport properties of microcrystalline silicon thin films prepared by Cat-CVD. Thin Solid Films.

[31] Ambrosone G, Coscia U, Cassinese A, Barra M, Restello S, Rigato V, Ferrero S (2007). Low temperature electric transport properties in hydrogenated microcrystalline silicon films. Thin Solid Films.

[32] Osinniy V, Lysgaard S, Kolkovsky V, Pankratov V, Larsen AN (2009). Vertical charge-carrier transport in Si nanocrystal/SiO2 multilayer structures. Nanotechnology.

